# Effect of hearing loss on cognitive function in patients with mild cognitive impairment: A prospective, randomized, and controlled study

**DOI:** 10.3389/fnagi.2022.934921

**Published:** 2022-08-01

**Authors:** Jie Tong, Jie Zhang, Luli Xu, Meiling Liu, Jie Min, Miaomiao Yao, Xiaoyan Cheng, Qi Zhang, Xirong Sun, Jie Yuan

**Affiliations:** ^1^Clinical Research Center for Mental Disorders, Shanghai Pudong New Area Mental Health Center, School of Medicine, Tongji University, Shanghai, China; ^2^Department of Otolaryngology, Shanghai Punan Hospital of Pudong New District, Shanghai, China

**Keywords:** hearing loss, cognitive function, mild cognitive impairment, MCI, HL

## Abstract

**Background:**

Hearing loss (HL) may increase the risk of cognitive decline in the elderly. However, the randomized controlled study on the effect of HL on cognitive function in mild cognitive impairment (MCI) is very limited.

**Methods:**

From 1 November 2020 to 30 March 2022, 1,987 individuals aged 55–65 years were randomly divided into the MCI with hearing impairment (MCI-HI), MCI without HI (MCI-nHI), and no MCI (nMCI) groups by stratified sampling, with 30 participants in each group. The Mini-Mental State Examination (MMSE), the Montreal Cognitive Assessment (MoCA), the pure tone audiometry (PTA), and the auditory brainstem response (ABR) were measured at baseline and a follow-up 12 months later. The trial protocol was registered with ClinicalTrials.gov with the registration number NCT05336942.

**Results:**

Among the 90 participants, the average age was 60.41 ± 6.48 years. In the MCI-HI group at baseline, the PTA score of both the ears was negatively correlated with the naming and memory score (*p* < 0.05), and the PTA score of both the ears was negatively correlated with the MoCA and abstraction score at the 12-month follow-up (*p* < 0.05). However, there were no significant differences among the PTA, the ABR, the MMSE, and the MoCA scores in the MCI-nHI and nMCI groups (*p* > 0.05). Regression analysis showed that the PTA score of the right ear at baseline was an important factor associated with the MoCA, visuospatial/executive, naming, and abstraction scores at the 12-month follow-up (β = −0.776 to −0.422, *p* < 0.05).

**Conclusion:**

HL was significantly negatively associated with cognitive function only in patients with MCI with hearing impairment (HI), and the PTA of the right ear may be a predictor of cognitive decline after 1 year in patients with MCI with HI. This information may help primary healthcare clinicians to prevent MCI by screening and intervening in care for elderly patients with HL.

## Background

More than 55 million people worldwide suffer from dementia, and this number is expected to increase to ~78 million affected people in 2030, with an estimated cost of US $2 trillion by 2050 (Jia et al., 2020; Gauthier et al., 2021). Mild cognitive impairment (MCI) is a symptomatic precursor stage of cognitive decline, and an intermediate state between dementia and normal cognitive function (Hill et al., 2017; Jongsiriyanyong and Limpawattana, 2018; Hemminghyth et al., 2020). The risk of Alzheimer's disease in patients with MCI is 10 times that of people with normal cognitive function, and MCI has become the most important risk factor for dementia (Serrano-Pozo and Growdon, 2019; Scheltens et al., 2021). A systematic review showed that the conversion rates from MCI to vascular dementia, Alzheimer's disease, and dementia were 6.2, 33.6, and 39.2%, respectively (Mitchell and Shiri-Feshki, 2009). Identifying modifiable risk factors for MCI will enable early intervention to prevent or substantially delay the onset of dementia (Vega and Newhouse, 2014; Sachs-Ericsson and Blazer, 2015).

The 2021 World Hearing Report shows that hearing loss (HL) affects more than 1.5 billion people worldwide, with more than 65% of those over 60 years of age having some degree of hearing loss (Chadha et al., 2021). Elderly people with hearing impairment (HI) are 2–5 times more likely to develop dementia than those with normal hearing (Griffiths et al., 2020). Most prospective cohort studies on the association between HL and Alzheimer's disease found that HL significantly increased the risk of Alzheimer's disease (Zheng et al., 2017; Llano et al., 2020, 2021). Some studies have also proposed age-related hearing loss (ARHL) as a possible non-invasive biomarker that predates the onset of clinical dementia by 5–10 years (Rutherford et al., 2018; Golub et al., 2019). Several potential mechanisms suggest that auditory deprivation may cause decreased socialization and affect cognitive function (Mick et al., 2014; Paciello et al., 2021). In addition, HL may cause cognitive resources to be diverted from memory function into auditory processing, which creates an excessive cognitive load on higher cortical functions (Pichora-Fuller et al., 2016; Van Canneyt et al., 2021).

However, few randomized controlled studies have focused on the effect of HL on MCI, and only a few cross-sectional studies have explored the association between HL and cognitive function. Lim and Loo (2018) conducted a cross-sectional study with a natural sample of 115 older adults and showed that HL is associated with MCI, but cognitive scoring may be confounded by poor hearing ability. The Mayo Clinic Study of Aging involving 4,812 participants found that participants with HL had a higher risk of MCI [hazard ratio (HR) 1.29, 95% CI 1.10–1.51], and considered that HL was associated with modestly greater cognitive decline (Vassilaki et al., 2019). Additionally, these studies did not separately describe left and right hearing functions in specific MCI samples. It is not known whether these differences relate to the link between HL and the risk of dementia. We attempted to use a community-based, multicenter natural sample to explore the long-term effects of left- and right-sided HL on cognitive function in patients with MCI by randomized, controlled, and longitudinal studies and compared the results with from normal hearing and normal cognitive populations. If a potential causal relationship between HL and MCI can be found, it will provide insights into the early prevention of cognitive impairment in clinical settings.

## Methods

### Study design

The study was designed as a prospective, randomized, and controlled survey. It is based on the Shanghai Pudong Mental Health Center (PMHC), Tongji University School of Medicine, which has been working on building a comprehensive cognitive impairment laboratory since 2008. Additionally, we cooperated with the hearing laboratory of Shanghai Punan Hospital in Pudong New District. The sample size of the study was calculated using the PASS version 21.0.3 (NCSS LLC, Utah, USA), a sample size, and power analysis software. The significance level was 0.05, a two-sided test was needed, and the power value was 0.8. The sample size of mild cognitive impairment with hearing impairment (MCI-HI), mild cognitive impairment without hearing impairment (MCI-nHI), and no mild cognitive impairment (nMCI) was estimated to be 27 participants per group. We set a 10% loss rate, including participants who could not complete the test or dropout due to special reasons, and finally determined a sample size of 30 participants for each group.

### Participants and randomization procedure

Five of 23 communities were randomly selected in Pudong New District, Shanghai, from 1 November 2020 to 30 March 2022. A total of 1,987 individuals aged 55–65 years were selected from the cognitive function database of the communities. Among them, there were 225 patients with MCI and 1,696 individuals without cognitive impairment. A total of 201 patients with MCI were willing to undergo rapid hearing screening (Path Medical handheld hearing screeners) and patients or their guardians signed the consent form. By stratified sampling, patients were divided into the three groups: the MCI-HI group, the MCI-nHI group, and the nMCI group, with 30 participants in each group.

The following inclusion criteria were employed: (1) patients meeting the Diagnostic and Statistical Manual of Mental Disorders-5 (DSM-5) (Roehr, 2013) diagnostic MCI; (2) 55 years old ≤ age ≤ 65 years old; (3) the Mini-Mental State Examination (MMSE) score ≤ 26 (Zhuang et al., 2021); (4) the pure tone audiometry (PTA) test score of the left or right ear ≥ 26 dB HL (Lapsley Miller et al., 2018); (5) normal or partially impaired ability to complete daily living activities; (6) ability to conduct verbal communication or written conversation; (7) capacity to complete the evaluation scale independently; and (8) participants or guardians agreed and signed the informed consent form for the study. The following exclusion criteria were employed: (1) patients meeting the DSM-5 (Roehr, 2013) diagnostic criteria of dementia, schizophrenia, neurosis, organic mental disorder, and intellectual disability; (2) severe extracranial trauma, limb disability, or physical illness; (3) those who were obviously blind or had difficulty in speech expression; (4) those who had perforated tympanic membrane perforation and used hearing aids previously; and (5) participants or guardians who did not sign the study informed consent or dropped out halfway.

## Measures

### Mini-mental state examination

The MMSE scale was proposed by Folstein et al. in 1975 (Folstein et al., 1975). It is widely used to measure cognitive impairment in clinical and research settings, including simple tasks in a number of areas: orientation, registration, attention, and calculation such as serial subtractions of seven, recall, and language. The Chinese version of the MMSE was created by Li et al. in 1989 and provides better reliability and validity. The Cronbach's α coefficient was 0.82 and the remeasuring reliability was 0.89 (Li et al., 1989). There are 30 items, with 1 point for the correctness and 0 points for error. Individuals with junior high school education and above had the MMSE ≤ 26, individuals with primary education had the MMSE ≤ 22, and individuals with no education had the MMSE ≤ 19, and were considered to have MCI (Zhang et al., 1999).

### Montreal cognitive assessment

The MoCA scale was developed based on the clinical intuition of impairment commonly encountered in MCI and is best adapted to a screening test. This 30-point test, which was introduced by Nasreddine et al. in 2005 (Nasreddine et al., 2005), covers eight cognitive domains. The Chinese version of the MoCA was culturally and linguistically modified by Lu et al. in 2011. The Cronbach's α coefficient was 0.82, and the remeasuring reliability was 0.86 (Lu et al., 2011). There are 12 items, and the total score ranges from 0 to 30. The MoCA score > 26 indicates normal cognitive function. The cutoff value is 25, if the length of education is ≤ 12 years.

### Pure tone audiometry

Pure tone audiometry testing is used to determine hearing threshold levels and to characterize the degree, and type of hearing loss. This test is a subjective and behavioral measurement of the hearing threshold (World Health Organization, 1999). The binaural (right ear first) air conductance hearing threshold was measured at 0.5, 1, 2, and 4 kHz. The average hearing threshold for normal hearing was defined as <25 dB HL (Louw et al., 2018).

### Auditory brainstem response

Auditory brainstem response testing, which is also known as brainstem auditory evoked potentials (BAEPs), is the electrical response of the auditory nerve and brainstem nucleus caused by acoustic stimulation. This test can be used to express the electrical activities of the cochlea, auditory nerve, and brainstem auditory pathway and objectively evaluate the threshold of auditory behavior (Laumen et al., 2016). After receiving 10 ms of short sound stimulation, seven vertex positive waves with negative valleys can be traced from the surface of the skull skin. Wave V is generated in the auditory brainstem, which has the highest amplitude. This test is often used as a clinical diagnostic criterion for hearing loss (Møller and Jannetta, 1982). The binaural (right ear first) brainstem response threshold was measured at 0.5, 1, 2, and 4 kHz, which is usually 10–20 dB higher than that of pure tone audiometry (Eggermont, 2019; McKearney et al., 2021).

### Procedure

With reference to the slopes of cognitive and hearing decline, all the enrolled participants received a cognitive function and hearing function at baseline and at a follow-up 12 months later (Kuo et al., 2021; Jang et al., 2022). Participants were instructed by an experimenter to assess the MMSE and the MoCA scales according to instructions in a quiet room, and to check the completion of each item. The evaluator has a Master's degree in psychiatry and is a registered cognitive function scale surveyor in China. Subsequently, we used the modern and versatile Eclipse platform produced by Interacoustics (Denmark), including a Melison AD104 diagnostic audiometer and AT235 automatic middle ear analyzer. Before the evaluation, the data and equipment of all the hearing test instruments were calibrated by the manufacturer. The hearing test was carried out in a professional pure tone electric audiometry room, and the environmental requirements met the *Chinese Basic Audiometry of Pure Tone Air Conductance and Bone Conductance Threshold* (GB/T 16296-2018) (indoor noise level: ≤ 30 dB; air exchange rate: 10 times per h; indoor temperature: 20–26°C; and humidity: 40–80% RH) (CNSIPSP, 2018). The evaluation was performed by a senior otolaryngology specialist with a Chinese registered hearing test certificate. All the evaluators were trained for consistency.

### Data analysis

Data were analyzed using the R Foundation for Statistical Computing (version 4.1.1) (R Software, 2021). We performed normality tests on all the data, using mean ± SD to statistically describe normal continuous data, median [interquartile range (IQR)] to statistically describe non-normal continuous data, and used the ANOVA or non-parametric rank-sum test for intergroup comparisons. For classified data, the frequency (percentage) was used for statistical description, and the chi-squared test/Fisher's exact probability method was used for intergroup comparison. Spearman's correlation analysis was used to evaluate the correlation between research indicators. Multiple linear regression analysis was performed to determine the association between hearing and cognitive functions. The difference was statistically significant at *p* < 0.05.

### Ethics statement

The protocol for this research was approved by the Research Ethics Committee of the Shanghai Pudong New Area Mental Health Center and Tongji University School of Medicine (No: PDJWLL2019017). All the procedures were performed in accordance with the ethical standards of the responsible committee on human experimentation (institutional and national) and the Declaration of Helsinki of 1975, as revised in 2008. The participants and guardians provided written informed consent to participate in this study. The trial protocol was registered with ClinicalTrials.gov with the registration number NCT05336942.

## Results

### Demographic characteristics

The demographic characteristics of participants from the MCI-HI, MCI-nHI, nMCI, and overall groups in terms of age, sex, education, occupation, marital status, living condition, family financial satisfaction, and self-rated health condition are shown in Table 1. Among the 90 participants, the average age was 60.41 ± 6.48 years, and the proportion of females was higher (55.56%) than males. In addition, most of the participants had a secondary school education (68.89%), were incumbent or retired (65.55%), were married (75.56%), lived with spouse or children (70.00%), were satisfied with family financial status (60.00%), and had ordinary self-rated health conditions (41.11%). There were significant differences in age and education among the three groups (*p* < 0.05), but there was no significant difference in other demographic variables (*p* > 0.05).

**Table 1:** Demographic characteristics of participants in each group.

**Variable**	**Overall (*n* = 90)**	**MCI-HI (*n* = 30)**	**MCI-nHI (*n* = 30)**	**nMCI (*n* = 30)**	***F*/χ2**	***p***
Age in years, (mean ± SD)	60.41 ± 6.48	63.63 ± 4.29	61.27 ± 5.87	56.33 ± 6.86	12.49	<0.001**
Sex, *n* (%)					2.52	0.284
Male	40 (44.44%)	10 (33.33%)	14 (46.67%)	16 (53.33%)		
Female	50 (55.56%)	20 (66.67%)	16 (53.33%)	14 (46.67%)		
Education, *n* (%)					9.48	0.035*
Primary school and below	22 (24.44%)	11 (36.67%)	7 (23.33%)	4 (13.33%)		
Secondary school	62 (68.89%)	15 (50.00%)	23 (76.67%)	24 (80.00%)		
College and above	6 (6.67%)	4 (13.33%)	0 (0.00%)	2 (6.67%)		
Occupation, *n* (%)					5.63	0.160
incumbent or retired	59 (65.55%)	18 (60.40%)	18 (60.00%)	23 (76.67%)		
Unemployed	31 (34.45%)	12 (40.00%)	12 (40.00%)	7 (23.33%)		
Marital status, *n* (%)					3.76	0.450
Unmarried	16 (17.78%)	4 (13.33%)	4 (13.33%)	8 (26.67%)		
Married	68 (75.56%)	25 (83.33%)	24 (80.00%)	19 (63.33%)		
Divorced or widowhood	6 (6.67%)	1 (3.33%)	2 (6.67%)	3 (10.00%)		
Living condition, *n* (%)					2.13	0.230
Living alone	27 (30.00%)	7 (23.33%)	8 (26.67%)	12 (40.00%)		
Living with spouse or children	63 (70.00%)	23 (76.67%)	22 (73.33%)	18 (60.00%)		
Family financial satisfaction, *n* (%)					5.03	0.085
Satisfied	54 (60.00%)	16 (53.33%)	18 (60.00%)	20 (66.67%)		
Dissatisfied	36 (40.00%)	14 (46.67%)	12 (40.00%)	10 (33.33%)		
Self-rated health condition, *n* (%)					6.81	0.053
Good	23 (25.56%)	8 (26.67%)	6 (20.00%)	9 (30.00%)		
Ordinary	37 (41.11%)	10 (33.33%)	14 (46.67%)	13 (43.33%)		
Bad	30 (33.33%)	12 (40.00%)	10 (33.33%)	8 (26.67%)		

MCI-HI, Mild cognitive impairment with hearing impairment; MCI-nHI, Mild cognitive impairment without hearing impairment; nMCI, No mild cognitive impairment; *p < 0.05, **p < 0.01.

### Hearing and cognitive functions

The hearing and cognitive functions among participants from the overall and the three groups at baseline and the 12-month follow-up are given in Table 2. Given the significant differences in age and education among the groups, after controlling for age and education as covariates, the differences in hearing and cognition functions among the groups were compared. In terms of hearing function, the PTA and ABR scores of the left and right ears were the highest in the MCI-HI group at baseline, and there were significant differences among the three groups (*p* < 0.001). Compared with the baseline, the PTA and ABR scores of both the ears increased in each group at the 12-month follow-up. However, there was no significant difference in the PTA scores in the MCI-HI group at the 12-month follow-up (*t* = −1.51 to 0.85, *p* > 0.05), while there were significant differences in the PTA and ABR scores in the other groups (*p* < 0.05). In terms of cognitive function, the MMSE and the MoCA scores in the nMCI group decreased at the 12-month follow-up, and there were significant differences (*z* = −1.76 to −0.94, *p* < 0.01). In the MCI-HI and MCI-nHI groups, the abstraction and memory scores of the MoCA decreased at the 12-month follow-up, and there were significant differences (*z* = −5.06 to −2.00, *p* < 0.01). There was no significant difference in the MMSE and the MoCA scores between the other groups (*p* > 0.05).

**Table 2:** Hearing and cognitive functions of participants in each group.

**Variable**	**Follow-up**	**Overall (*n* = 90)**	**MCI-HI (*n* = 30)**	**MCI-nHI (*n* = 30)**	**nMCI (*n* = 30)**	***F*/χ2**	***p***
**PTA, (mean** **±SD)**
Left	B/L	26.22 ± 10.34	37.41 ± 10.17	18.86 ± 4.70	22.40 ± 1.48	68.36	<0.001**
	12M	33.81 ± 9.05	40.93 ± 7.72	27.84 ± 7.43	33.67 ± 5.84	30.03	<0.001**
	*t* (*p*)	−5.24 (<0.001**)	−1.51 (0.136)	−4.98 (<0.001**)	−10.24 (<0.001**)		
Right	B/L	25.74 ± 9.58	35.79 ± 9.93	20.62 ± 4.76	20.82 ± 1.93	54.46	<0.001**
	1 M	34.41 ± 8.76	37.83 ± 8.61	31.22 ± 8.06	32.18 ± 8.59	4.63	0.012*
	*t* (*p*)	−6.33 (<0.001**)	−0.85(0.398)	−6.20 (<0.001**)	−8.31 (<0.001**)		
**ABR, (mean** **±SD)**
Left	B/L	30.00 (10.00)	42.50 (15.00)	27.50 (5.00)	25.00 (8.00)	44.81	<0.001**
	12M	40.00 (20.00)	50.00 (15.00)	32.50 (11.00)	40.00 (11.00)	26.56	<0.001**
	*z* (*p*)	−5.47 (<0.001**)	−2.72 (0.006**)	−3.80 (<0.001**)	−5.10 (<0.001**)		
Right	B/L	30.00 (15.00)	40.00 (11.00)	27.50 (11.00)	25.00 (5.00)	46.74	<0.001**
	12M	40.00 (15.00)	50.00 (16.00)	35.00 (11.00)	40.00 (10.00)	29.67	<0.001**
	*z* (*p*)	−5.78 (<0.001**)	−2.55 (0.011*)	−3.59 (<0.001**)	−5.99 (<0.001**)		
MMSE, (mean ± SD)	B/L	25.00 (3.00)	24.00 (2.00)	24.00 (2.00)	28.50 (2.00)	58.43	<0.001**
	12M	25.00 (4.00)	23.50 (2.00)	24.00 (2.00)	27.50 (3.00)	53.12	<0.001**
	*z* (*p*)	−1.61 (0.108)	−1.89 (0.058)	−1.17 (0.243)	−0.94 (0.002**)		
MoCA, (mean ± SD)	B/L	23.00 (4.00)	22.00 (5.00)	23.00 (3.00)	26.00 (5.00)	22.71	<0.001**
	12M	22.00 (3.00)	21.00 (5.00)	22.00 (3.00)	24.00 (4.00)	23.716	<0.001**
	*z* (*p*)	−2.28 (0.022*)	−1.74 (0.082)	−1.03 (0.303)	−1.76 (0.001**)		
Visuospatial/executive	B/L	3.00 (1.00)	4.00 (2.00)	3.00 (0.00)	4.00 (2.00)	12.11	0.002**
	12M	3.00 (2.00)	2.00 (2.00)	3.00 (1.00)	3.00 (2.00)	6.769	0.034*
	*z* (*p*)	−2.60 (0.009**)	−1.20 (0.230)	−1.59 (0.112)	−2.27 (0.001**)		
Naming	B/L	3.00 (1.00)	3.00 (0.00)	2.50 (1.00)	3.00 (1.00)	13.72	0.001**
	12M	3.00 (0.00)	3.00 (1.00)	3.00 (0.00)	3.00 (0.00)	1.65	0.438
	*z* (*p*)	−1.12 (0.261)	−0.62 (0.538)	−1.98 (0.047*)	−1.22 (0.222)		
Attention	B/L	5.00 (1.00)	5.00 (1.00)	5.00 (0.00)	6.00 (1.00)	22.78	<0.001**
	12M	5.00 (2.00)	4.50 (1.00)	4.00 (1.00)	6.00 (0.00)	32.225	<0.001**
	*z* (*p*)	−0.95 (0.342)	−0.34 (0.736)	−2.99 (0.003**)	−0.89 (0.371)		
Language	B/L	2.00 (1.00)	2.00 (1.00)	2.00 (0.00)	3.00 (0.00)	17.38	<0.001**
	12M	2.00 (1.00)	2.00 (1.00)	2.00 (1.00)	3.00 (1.00)	5.18	0.075
	*z* (*p*)	−1.35 (0.178)	−1.03 (0.303)	−0.662 (0.508)	−1.93 (0.054)		
Abstraction	B/L	1.00 (1.00)	1.00 (2.00)	1.00 (1.00)	2.00 (1.00)	16.47	<0.001**
	12M	2.00 (0.00)	2.00 (1.00)	2.00 (0.00)	2.00 (0.00)	4.53	0.104
	*z* (*p*)	−5.07 (<0.001**)	−3.68 (<0.001**)	−5.06 (<0.001**)	−0.43 (0.671)		
Memory	B/L	3.00 (2.00)	2.50 (1.00)	2.50 (2.00)	3.00 (2.00)	0.36	0.837
	12M	2.00 (2.00)	1.00 (2.00)	2.00 (1.00)	3.00 (3.00)	15.44	<0.001**
	*z* (*p*)	−3.49 (<0.001**)	−3.95 (<0.001**)	−2.00 (0.045*)	−0.55 (0.583)		
Orientation	B/L	6.00 (0.00)	6.00 (1.00)	6.00 (0.00)	6.00 (0.00)	9.87	0.007**
	12M	6.00 (0.00)	6.00 (1.00)	6.00 (0.00)	6.00 (0.00)	7.62	0.022*
	*z* (*p*)	−0.25 (0.803)	−0.115 (0.909)	−1.00 (0.317)	−0.014 (0.989)		

MCI-HI, Mild cognitive impairment with hearing impairment; MCI-nHI, Mild cognitive impairment without hearing impairment; nMCI, No mild cognitive impairment; MMSE, Mini-Mental State Examination; MoCA, Montreal Cognitive Assessment (The Montreal Cognitive Assessment, MoCA: a brief screening tool for mild cognitive impairment), PTA, Pure tone audiometry, ABR, Auditory brainstem response; B/L, Baseline; 12M, 12-month; *p < 0.05, **p < 0.01.

### Correlation analysis

**Figure 1:**
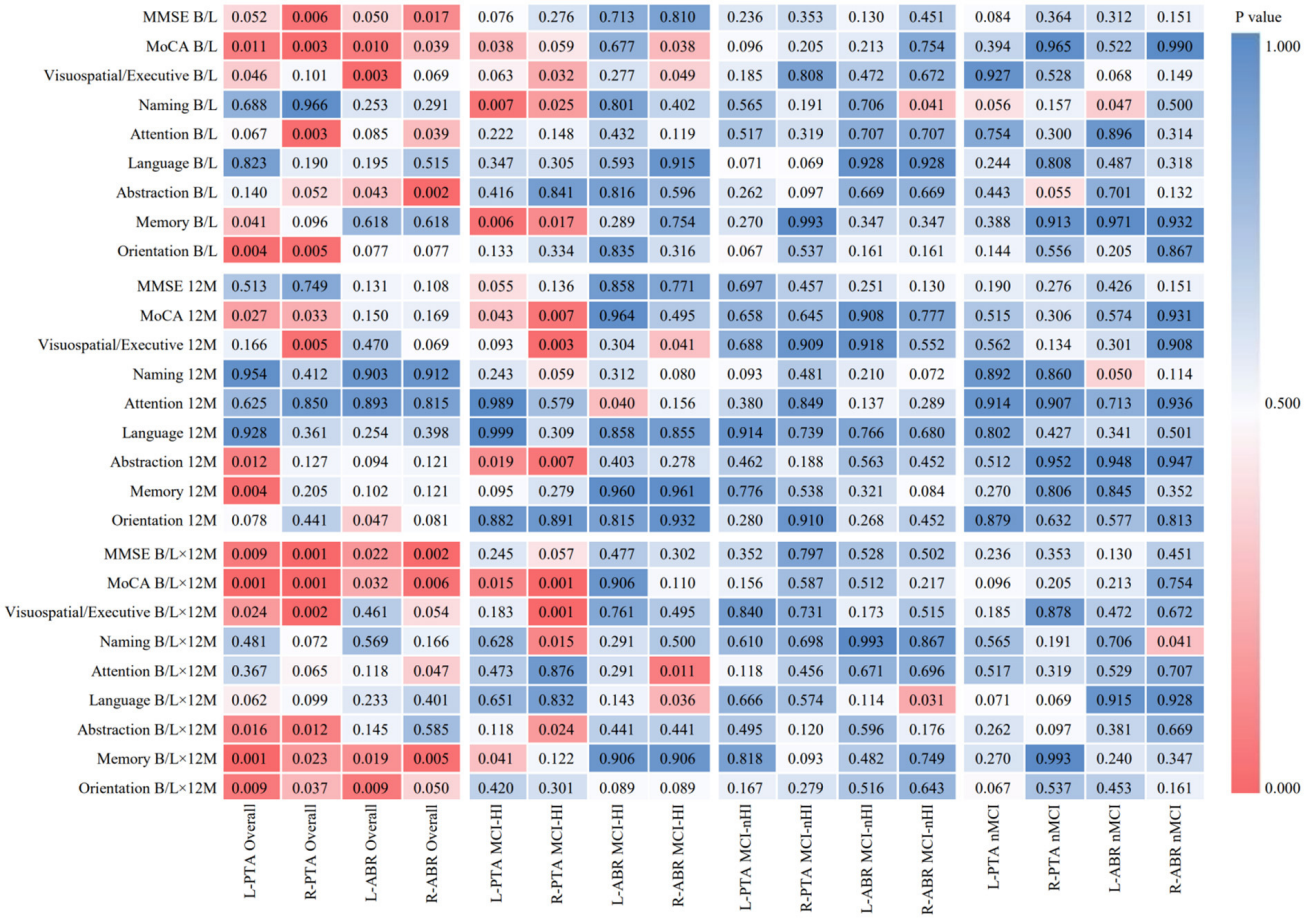
Heat map of correlation analysis between hearing and cognitive functions in different periods. MCI-HI, Mild cognitive impairment with hearing impairment; MCI-nHI, Mild cognitive impairment without hearing impairment; nMCI, No mild cognitive impairment; MMSE, Mini-Mental State Examination; MoCA, Montreal Cognitive Assessment (The Montreal Cognitive Assessment, MoCA, a brief screening tool for mild cognitive impairment), PTA, Pure tone audiometry, ABR, Auditory brainstem response; B/L, Baseline; 12M, 12-month; B/L×12 M, Baseline PTA and ABR scores × 12-month MMSE and MOCA scores.

Spearman's correlation analysis was conducted on the PTA and ABR of the left and right ears, the MMSE, and eight dimensions of the MoCA among participants from the overall and three groups, as shown in Figure 1. In the MCI-HI group at baseline, the PTA score of both the ears was negatively correlated with the naming and memory scores (*p* < 0.05), and the ABR score of the right ear was negatively correlated with the MoCA and visuospatial/executive scores (*p* < 0.05). At the 12-month follow-up, the PTA scores of both the ears were negatively correlated with the MoCA and abstraction scores (*p* < 0.05). Meanwhile, the PTA scores of both the ears at baseline were negatively correlated with the MoCA scores at the 12-month follow-up (*p* < 0.05), and the ABR scores of the right ear at baseline were negatively correlated with the attention and language scores at the 12-month follow-up (*p* < 0.05). However, there were no significant differences between the PTA, ABR, the MMSE, and the MoCA scores in the MCI-nHI and nMCI groups (*p* > 0.05).

### Regression analysis

In the MCI-HI group, the independent variables were the PTA and ABR scores of both the ears; the MMSE and the MoCA scores were the dependent variables for multiple linear regression analysis. The PTA score of the right ear at baseline was an important factor associated with the MoCA, visuospatial/executive, naming, and abstraction scores at 12-month follow-up (β = −0.776 to −0.422, *p* < 0.05). At the baseline, the ABR scores of both the ears were an important factor associated with the attention score (β = −0.794 to 0.781, *p* < 0.01) (Table 3).

**Table 3:** Multiple linear regression analysis of hearing and cognitive functions in the MCI-HI group.

**Variable**	**Follow-up**	**PTA**								**ABR**							
		**Left**				**Right**				**Left**				**Right**			
		**β**	***t***	***p***	**VIF**	**β**	***t***	***p***	**VIF**	**β**	***t***	***p***	**VIF**	**β**	***t***	***p***	**VIF**
MMSE	B/L	−0.347	−1.480	0.151	1.618	−0.120	−0.557	0.582	1.374	0.203	0.699	0.491	2.494	0.006	0.019	0.985	2.596
	12M	−0.569	−2.018	0.054	2.656	−0.114	−0.506	0.617	1.679	0.475	1.481	0.151	3.441	−0.080	−0.260	0.797	3.130
	B/L ×12M	−0.032	−0.139	0.891	1.618	−0.239	−1.126	0.271	1.374	0.328	1.149	0.261	2.494	−0.402	−1.378	0.180	2.596
MoCA	B/L	−0.178	−0.831	0.414	1.374	−0.178	−0.831	0.414	1.374	0.347	1.204	0.240	2.494	−0.286	−0.970	0.341	2.596
	12M	−0.389	−1.500	0.146	2.656	−0.400	−1.941	0.064	1.679	0.440	1.493	0.148	3.441	−0.078	−0.278	0.783	3.130
	B/L ×12M	−0.004	−0.019	0.985	1.618	−0.448	−2.290	0.031*	1.374	0.176	0.669	0.510	2.494	−0.296	−1.100	0.282	2.596
Visuospatial/executive	B/L	0.036	0.160	0.874	1.618	−0.347	−1.660	0.109	1.374	−0.052	−0.184	0.856	2.494	−0.210	−0.730	0.472	2.596
	12M	0.052	0.190	0.851	2.656	−0.413	−1.880	0.072	1.679	0.243	0.775	0.446	3.441	−0.389	−1.299	0.206	3.130
	B/L ×12M	0.297	1.637	0.114	1.618	−0.776	−4.643	0.000**	1.374	−0.003	−0.013	0.990	2.494	−0.063	−0.276	0.785	2.596
Naming	B/L	0.288	1.253	0.222	1.618	0.208	0.981	0.336	1.374	−0.065	−0.229	0.821	2.494	0.044	0.151	0.881	2.596
	12M	−0.114	−0.382	0.706	2.656	−0.118	−0.498	0.623	1.679	0.204	0.599	0.554	3.441	−0.386	−1.190	0.245	3.130
	B/L ×12M	0.019	0.096	0.925	1.618	−0.597	−3.258	0.003**	1.374	0.066	0.266	0.792	2.494	0.316	1.255	0.221	2.596
Attention	B/L	−0.056	−0.270	0.789	1.618	−0.009	−0.046	0.964	1.374	0.781	3.014	0.006**	2.494	−0.794	−3.002	0.006**	2.596
	12M	−0.423	−1.526	0.140	2.656	−0.128	−0.583	0.565	1.679	0.650	2.061	0.050	3.441	0.044	0.145	0.886	3.130
	B/L ×12M	0.006	0.029	0.977	1.618	0.155	0.754	0.458	1.374	0.321	1.159	0.257	2.494	−0.705	−2.498	0.019*	2.596
Language	B/L	0.044	0.182	0.857	1.618	0.277	1.254	0.221	1.374	0.367	1.234	0.229	2.494	−0.359	−1.184	0.247	2.596
	12M	−0.010	−0.032	0.975	2.656	−0.310	−1.268	0.216	1.679	0.151	0.430	0.671	3.441	−0.094	−0.280	0.782	3.130
	B/L ×12M	0.213	0.948	0.352	1.618	−0.055	−0.266	0.792	1.374	−0.199	−0.714	0.482	2.494	−0.368	−1.293	0.208	2.596
Abstraction	B/L	−0.180	−0.738	0.467	1.618	−0.039	−0.172	0.865	1.374	−0.273	−0.900	0.377	2.494	0.414	1.337	0.193	2.596
	12M	−0.348	−1.315	0.200	2.656	−0.420	−1.995	0.057	1.679	0.091	0.301	0.766	3.441	0.129	0.448	0.658	3.130
	B/L ×12M	−0.162	−0.726	0.474	1.618	−0.422	−2.058	0.048*	1.374	−0.117	−0.424	0.675	2.494	0.357	1.266	0.217	2.596
Memory	B/L	−0.522	−2.647	0.014*	1.618	−0.250	−1.376	0.181	1.374	0.315	1.288	0.210	2.494	0.114	0.458	0.651	2.596
	12M	−0.555	−1.916	0.067	2.656	−0.124	−0.539	0.595	1.679	0.239	0.724	0.476	3.441	0.240	0.763	0.453	3.130
	B/L ×12M	−0.378	−1.626	0.116	1.618	−0.103	−0.482	0.634	1.374	0.139	0.481	0.634	2.494	−0.003	−0.009	0.993	2.596
Orientation	B/L	0.073	0.305	0.763	1.618	−0.355	−1.611	0.120	1.374	−0.041	−0.139	0.891	2.494	−0.045	−0.149	0.883	2.596
	12M	−0.072	−0.224	0.825	2.656	0.052	0.205	0.840	1.679	0.230	0.628	0.536	3.441	−0.265	−0.760	0.454	3.130
	B/L ×12M	−0.019	−0.076	0.940	1.618	−0.022	−0.094	0.926	1.374	−0.013	−0.043	0.966	2.494	−0.171	−0.542	0.593	2.596

MCI-HI, Mild cognitive impairment with hearing impairment; PTA, Pure tone audiometry, ABR, Auditory brainstem response; MMSE, Mini-Mental State Examination; MoCA, Montreal Cognitive Assessment; B/L, Baseline; 12M, 12-month; B/L ×12M, Baseline PTA and ABR scores × 12-month MMSE and MOCA scores; *p < 0.05, **p < 0.01.

## Discussion

We measured the PTA, ABR, the MMSE, and the MoCA in community-based patients with MCI through a randomized, controlled, and longitudinal study to explore the correlation and long-term effect of HL and cognitive function in patients with MCI. To the best of our knowledge, this is the first study to focus on the effect of HL on cognitive function in patients with MCI by means of a randomized controlled study rather than by a cross-sectional survey, which is an original research direction. We found that HL was significantly negatively associated with cognitive function only in patients with MCI with HI, and was more significantly associated with cognitive function 1 year later. Meanwhile, the PTA of the right ear may be a predictor of cognitive decline after 1 year in patients with MCI with HI.

An English study of aging involving 14,767 adults aged 50 years and older showed that participants with self-reported or objective moderate and poor hearing were more likely to be diagnosed with dementia than those with normal hearing (Davies et al., 2017). A cross-sectional study of 995 Japanese adults aged 36–84 years suggested that HI was independently associated with a higher prevalence of MCI in elderly adults aged 60–69 and 70 years or older (Miyake et al., 2020). These findings are partially consistent with our results, but these studies did not explain the association of cognitive reserve with hearing loss and cognitive function. Chen and Lu (2020) found that hearing-impaired elderly with low cognitive reserve had the highest risk of cognitive impairment [odds ratio (OR) 4.32, 95% CI 3.42–5.47], further confirming that cognitive reserve moderated the negative association between hearing difficulties and cognitive function.

Some longitudinal studies of older adults have also confirmed the long-term effects of HI on cognitive function. In a 10-year cohort study conducted in the US, HI in patients and PTA > 25 dB were significantly positively associated with a 10-year risk of cognitive impairment in dementia or Alzheimer's disease (Fischer et al., 2016). In a meta-analysis of 15,521 subjects followed-up for 2–16.8 years, HI was associated with a higher risk of MCI [relative risk (RR) = 1.30, 95% CI: 1.12, 1.51] and dementia (RR = 2.39, 95% CI: 1.58, 3.61) (Wei et al., 2017). The Taiwan Longitudinal Study on Aging (TLSA) with a mean follow-up of 8.9 ± 3.9 years showed that HL was an independent risk factor for cognitive impairment other than geriatric syndromes (Tai et al., 2021). However, these studies did not group patients by the presence or absence of HI and were unable to show the association of HI with cognitive function in MCI subgroups.

There are also many different views on the impact of binaural hearing differences on cognitive function. A controlled study of patients with tinnitus and normal adults showed that the tinnitus group performed significantly worse in the left ear than in the right ear, and this interaural difference may be influenced by a right-ear advantage for speech sounds, possibly interacting with cognitive factors (Tai and Husain, 2018). In the PTA and brain MRI study of 982 older adults, mild right ear HL in older women was associated with left frontal and bilateral occipital cortical thinning and mild-to-severe right ear HL was associated with bilateral frontal, right temporal, and bilateral occipital cortical thinning (Ha et al., 2020). A controlled study of 400 right-handed participants showed a right ear advantage in auditory processing, possibly corresponding to a left hemispheric advantage in verbal and nonverbal imagery (Prete et al., 2016). This also confirms our findings that hearing in the right ear is associated with areas of brain auditory feedback and cognitive function dominance, and that HL in the right ear compared to the left ear may be a predictor for the early identification of MCI. Additionally, it may help primary healthcare clinicians to prevent MCI by screening and intervening in elderly patients with HL.

## Limitations

We also note several limitations. First, research has been greatly limited with respect to expanding the number of samples because of the COVID-19 pandemic, and the current sample group is limited to individuals aged 55–65 years. Second, we did not extend brain imaging tests such as functional MRI (fMRI) or diffusion tensor imaging (DTI) to elucidate the relationship between HL, cognitive decline, and structural or functional features of the brain (Wang et al., 2021). Future studies should further explore whether the intervention of HL can reduce the risk of MCI in elderly patients, expand the study with 2-, 5- and 10-year follow-up periods, and observe the final outcome of the impact of HL on cognitive function. These studies would provide the exact mechanism to achieve an optimal effect in the early identification of MCI.

## Conclusion

In this study, HL was significantly negatively associated with cognitive function only in patients with MCI with HI, and the PTA of the right ear may be a predictor of cognitive decline after 1 year in patients with MCI with HI. This information may help primary healthcare clinicians to prevent MCI by screening and intervening in care for elderly patients with HL.

## Data availability statement

The original contributions presented in the study are included in the article/supplementary material, further inquiries can be directed to the corresponding author/s.

## Ethics statement

The studies involving human participants were reviewed and approved by the Research Ethics Committee of the Shanghai Pudong New Area Mental Health Center and Tongji University School of Medicine (No: PDJWLL2019017). The patients/participants provided their written informed consent to participate in this study. Written informed consent was obtained from the individual(s) for the publication of any potentially identifiable images or data included in this article.

## Author contributions

JT, JZ, and JY: data analysis and writing—original draft preparation and revising. XS and JY: conceptualization and writing—reviewing and editing. LX: hearing function testing, analysis, and interpretation. JT and JZ: supervision. JM: project administration. ML, MY, XC, and QZ: sample collection. All authors have approved the submitted version of the manuscript.

## Funding

This study was supported by grants from the following institutions: (1) the Science and Technology Development Fund of Shanghai Pudong New Area (No. PKJ2019-Y24) and (2) the Outstanding Clinical Discipline Project of Shanghai Pudong (Funding No: PWYgy2021-02).

## Conflict of interest

The authors declare that the research was conducted in the absence of any commercial or financial relationships that could be construed as a potential conflict of interest.

## Publisher's note

All claims expressed in this article are solely those of the authors and do not necessarily represent those of their affiliated organizations, or those of the publisher, the editors and the reviewers. Any product that may be evaluated in this article, or claim that may be made by its manufacturer, is not guaranteed or endorsed by the publisher.
